# Bacterial MerR family transcription regulators: activationby distortion: The mechanism of transcription regulation by MerR

**DOI:** 10.3724/abbs.2021003

**Published:** 2021-12-02

**Authors:** Chengli Fang, Yu Zhang

**Affiliations:** 1 Key Laboratory of Synthetic Biology CAS Center for Excellence in Molecular Plant Sciences Shanghai Institute of Plant Physiology and Ecology Chinese Academy of Sciences Shanghai 200032 China; 2 University of Chinese Academy of Sciences Beijing 100049 China

**Keywords:** RNA polymerase, gene transcription, gene expression, transcription factor, MerR

## Abstract

Transcription factors (TFs) modulate gene expression by regulating the accessibility of promoter DNA to RNA polymerases (RNAPs) in bacteria. The MerR family TFs are a large class of bacterial proteins unique in their physiological functions and molecular action: they function as transcription repressors under normal circumstances, but rapidly transform to transcription activators under various cellular triggers, including oxidative stress, imbalance of cellular metal ions, and antibiotic challenge. The promoters regulated by MerR TFs typically contain an abnormal long spacer between the –35 and –10 elements, where MerR TFs bind and regulate transcription activity through unique mechanisms. In this review, we summarize the function, ligand reception, DNA recognition, and molecular mechanism of transcription regulation of MerR-family TFs.

## Introduction

Unlike in eukaryotes, where the labor of gene transcription is split to three DNA-dependent RNA polymerases, *i*. *e*., polymerases I, II, and III [1], the gene transcription in bacteria solely replies on the single DNA-dependent RNA polymerase (RNAP) [2]. The bacterial RNAP partners with a set of transcription initiation factors (named σ factors) to form RNAP holoenzymes that are responsible for transcription of distinct gene programs [3]. In addition, a bacterial genome encodes hundreds of transcription factors (TFs) (~ 6% of their total gene count), which respond to environmental and cellular signals through their signal-reception domain (or ligand-binding domain) and modulate transcription of genes by directly binding to promoter DNA through their DNA-binding domain [4].

Bacterial transcription repressors occupy core promoter regions and inhibit transcription through preventing RNAP from engaging with promoter DNA, while the mechanism of bacterial transcription activation is much more complicated than that of transcription repression. The canonical Class I and Class II transcription activation models suggest that transcription activators interact with both RNAP and the upstream of core promoter region to increase local enrichment of RNAP at their regulated gene promoters and/or to facilitate subsequent promoter unwinding process [ 5, 6]. Distinct from the canonical transcription repression/activation models, the MerR-family TFs occupy the core promoter region to either repress or activate transcription of downstream genes depending on the cellular signals [ 7, 8].

In this review, we summarize the physiological function, ligand reception, DNA recognition, and mechanism of transcription regulation of MerR-family TFs. Recent comprehensive reviews are recommended for readers who are interested in mechanism of bacterial transcription and general bacterial transcription regulation [ 5, 6, 9, 15].

## Transcription Initiation by Bacterial RNAP

Most bacterial RNAP core enzymes are composed of five subunits, two identical α subunits, one β subunit, one β′ subunit, and one ω subunit [16]. The ω subunit is absent in certain bacterial species and additional small accessory subunits are gained in specific bacterial phyla [17]. The bacterial RNAP holoenzyme is composed of the RNAP core enzyme and one of σ factors which anchor their DNA-recognition domains (σ _2_, σ _3.1_, and σ _4_ of σ ^70^-type factors; region I and III for σ ^54^-type factors) on the surface of RNAP core enzyme and thread the linker domain (σ _3.2_ of σ ^70^-type factors; region II for σ ^54^-type factors) into the active-site cleft [ 18– 23].

**Figure 1:**
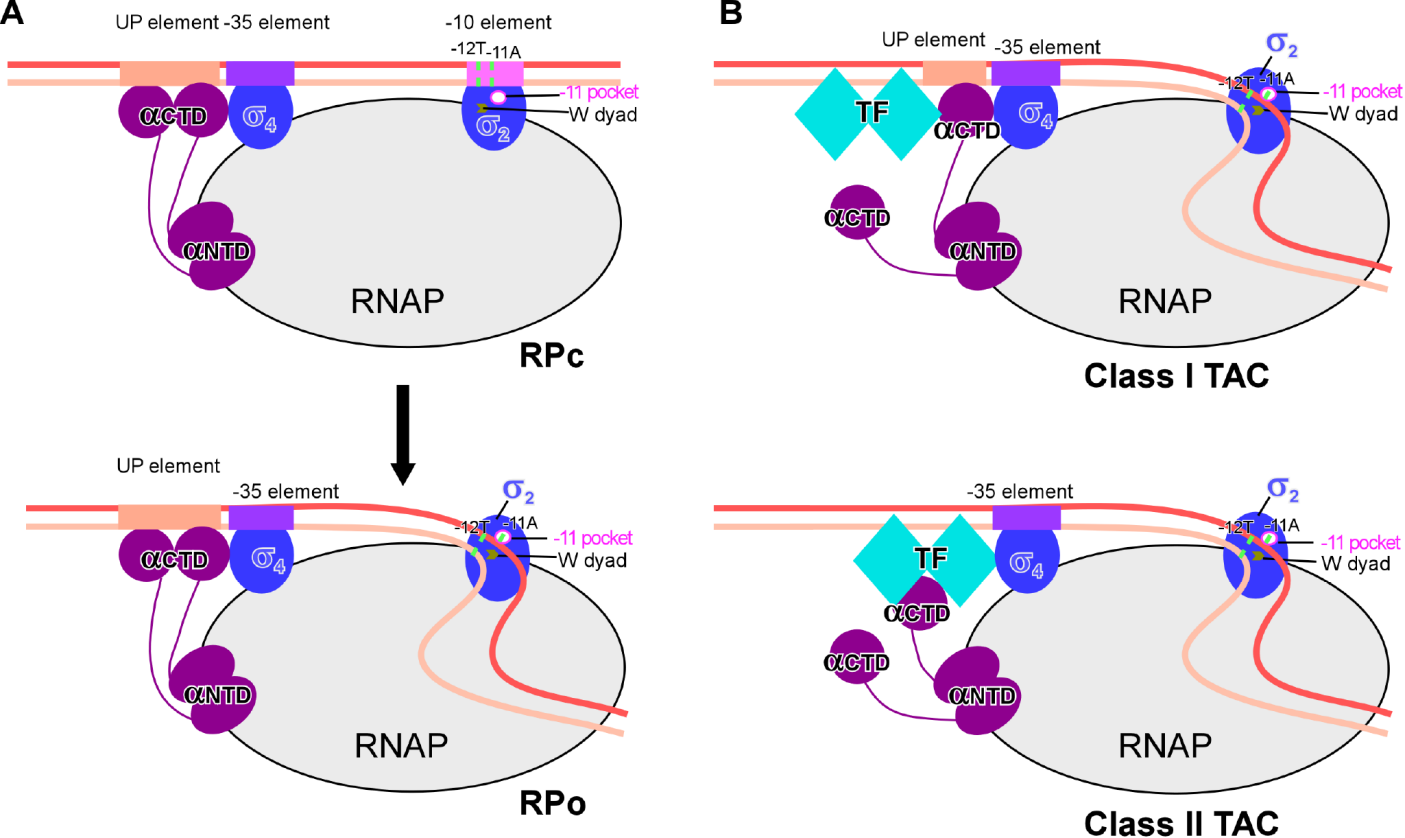
Figure1 **Models for bacterial transcription initiation and canonical transcription activation**(A) The model of RNAP-promoter DNA closed complex (RPc; upper panel) and RNAP-promoter DNA open complex (RPo; lower panel). In RPc, the double-stranded –35 element is recognized by σ4 in a sequence-specific manner, while the double-stranded –10 element is presented onto the surface of σ2 and restrained by sequence nonspecific electrostatic attraction interactions. In RPo, a ~13 bp transcription bubble is unwound and stabilized inside of RNAP. The base pair of –11 position is forced open by the W-dyad (W433 and W434 in E. coli RNAP σ70), and the adenine base of –11A of the non-template strand is recognized and secured in the –11 pocket. The other domains of σ factors are hidden for clarity. (B) The models for Class I (upper panel) and Class II (lower panel) transcription activation. A Class I transcription activator binds to the upstream of core promoter region and makes interactions with the C-terminal domain of the RNAP-α subunit (RNAP-αCTD). A Class II transcription activator binds to the proximal upstream of the core promoter region and makes interactions with both the RNAP-αCTD and σ4.

During transcription initiation, a RNAP-σ holoenzyme first recognizes the distal end of double-stranded core promoter DNA, *i*. *e*., the –35 element and/or extended –10 element, through sequence-specific interaction with σ _4_ and/or σ _3.1_ [ 24– 26]. The engagement of the distal end of promoter DNA presents its proximal end, *i*. *e*., the –10 element, on the surface of σ _2_, where the DNA unwinding initiates [ 27, 28]. The σ _4_/σ _2_ distance and the –35/–10 spacer length (optimally 17 base pairs; bp) are matched to allow the –35 and –10 elements to be recognized by σ _4_ and σ _2_ in concert [ 24, 29]. At the beginning of DNA unwinding, the base pair at the –11 position of the –10 element is disrupted and the most-conserved adenosine at the –11 position of non-template strand is flipped out and secured by a pocket of σ _2_ domain [ 28, 30, 31]. The DNA unwinding subsequently propagates to the downstream of promoter DNA and the unwound region of promoter DNA is stabilized in the main cleft of RNAP through base-specific pocket recognition at specific positions (T _–7_, G _–6_, and G _+2_ of the non-template strand) and electrostatic attraction interactions with the phosphate backbone [ 30, 32]. The resulting RNAP-promoter open complex (RPo) containing a ~13 bp unwound transcription bubble is competent for primer-dependent initiation (using a short RNA primer typically in length of 2–5 nt and one initiating NTP) or *de novo* initiation (using two initiating NTPs) of RNA synthesis ( Figure 1A) [ 24, 27, 29, 33– 35].

## Class I and Class II Transcription Activation

The *E*. *coli* catabolic-activated protein (CAP), also named cAMP-responsive protein (CRP), servers as the prototype of bacterial transcription factor for studying the molecular mechanism of transcription activation [ 5, 36]. The mode of transcription activation by CAP could be divided into two major classes based on the locations of CAP-binding *cis* element (CAP box) on promoter DNA. In the Class I mode of transcription activation, the CAP box is located at the upstream of core promoter region on the same face of the DNA helix as the UP and –35 elements (for example, the CAP boxes are usually centered at positions –62, –72, –83, and –93 of promoter DNA) [ 37, 38]. The *E*. *coli* CAP dimer, which binds to the CAP box at these positions, bends the upstream promoter towards RNAP to establish interaction with RNAP-α C-terminal domain (CTD) in the cryo-EM structure of *E*. *coli* CAP Class I transcription activation complex [ 39, 40]. The Class II CAP box is located at the proximal upstream of core promoter DNA, *i*. *e*., the CAP box is centered at position –41.5, that partially overlaps with the –35 element [ 38, 41]. The crystal structure of *T*. *thermophilus* TAP (a homolog of *E*. *coli* CAP) Class II transcription activation complex shows that the DNA-bound TAP makes interactions with both RNAP-α CTD and the σ _4_ domain [42]. Despite differences in location of *cis* elements and contact regions on RNAP, transcription factors bind to the upstream of promoter DNA and make bipartite interactions with promoter DNA and RNAP in both Class I and Class II transcription activation models ( Figure 1B) [ 5, 6, 36, 37].

## The MerR-family TFs

**Figure 2:**
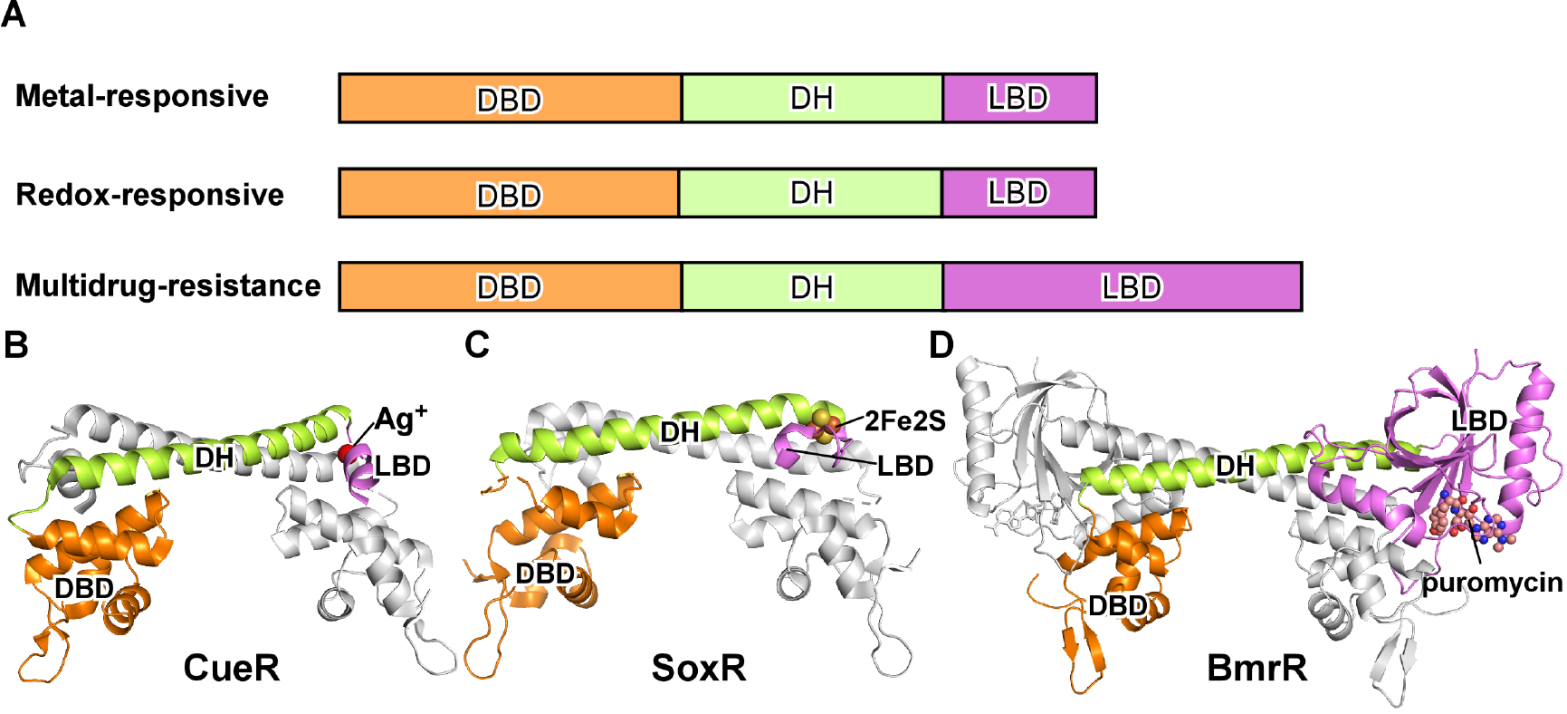
Figure2 **The MerR-family TFs**(A) The schematic of three categories of MerR-family TFs. DBD, DNA-binding domain; DH, dimerization helix; LBD, ligand-binding domain. (B) The structure of E. coli CueR dimer, a representative member of the metal-responsive MerR TFs, adapted from the crystal structure of Ag+-bound E. coli CueR-DNA complex (PDB: 4WLW). One protomer is colored in gray, and the second protomer is colored in orange (DBD), green (DH), and pink (LBD). The Ag+ is shows in gray sphere. (C) The structure of E. coli SoxR dimer, a representative member of the redox-responsive MerR TFs, adapted from the crystal structure of oxidated SoxR-DNA complex (PDB: 2ZHG). The [2Fe-2S] cluster is shown as sphere. (D) The structure of B. subtilis BmrR dimer, a representative member of the multidrug-resistance MerR TFs, adapted from the crystal structure of puromycin-bound BmrR-DNA complex (PDB: 1EXI). The puromycin is shown as sphere.

MerR-family TFs are a large family of bacterial TFs that share unique structural and mechanistic features. They typically contain an N-terminal DNA-binding domain (DBD), a central dimerization helix (DH), and a C-terminal ligand-binding domain (LBD) ( Figure 2A) [7]. Like most bacterial TFs, MerR-family TFs function as dimers. The two protomers interact with each other in a ‘head-to-head’ manner. The dimer interface is mainly contributed by the coiled-coil interaction of the central dimerization helices and inter-protomer DBD-LBD interaction ( Figure 2B). The MerR-family TFs could be further categorized into three subfamilies based on their physiological functions: the metal-responsive MerR-family TFs, the redox-responsive MerR-family TFs, and the multidrug-resistance MerR-family TFs.

### The metal-responsive MerR-family TFs

The metal-responsive MerR-family TFs contain members that specifically recognize various metal cations with +1 charge, for example Cu ^+^, Ag ^+^, and Au ^+^, or +2 charge, for example Cd ^2+^, Zn ^2+^, Pb ^2+^, Ni ^2+^, and Co ^2+^ ( Table 1). These patrol metal sensors rapidly respond to the elevated cellular ion concentration and subsequently ignite defense programs by activating expression of their regulated operons. For example, MerR, the founding member of MerR-family TFs, regulates the expression of the mercury-resistance operons that encode an enzyme catalyzing reduction of Hg ^2+^ to volatile Hg (MerA), transcription factors (MerD and MerR), a Hg ^2+^ scavenging protein Hg ^2+^ (MerP), and Hg ^2+^ transporters [ 43– 47]. The *R*. *metallidurans*PbrR regulates expression of the Pb ^2+^-resistance operon *pbrABCD*that encodes a Pb(II) efflux transporter (PbrA), a Pb(II)-binding protein(PbrD), and two function unknown proteins (PbrB and PbrC) [ 48, 49]. The *P*. *putida* CadR maintains cellular concentration of Cd ^2+^ by controlling the expression of a cadmium transporter (CadA) [ 50, 51].

**Table:** **Table1** The summary of representative MerR-family TFs

Regulator	Ligand	Organism	Regulated gene	Reference
*Metal-responsive MerR-family TFs*
MerR	Hg ^+^	Tn *21*/Tn *501*	Mercury-resistance operon *merTP (C*/ *F)AD(E)*	[ 52– 58]
ZntR	Zn ^2+^, Cd ^2+^, Pb ^2+^	*E*. *coli*	*zntA*, a metal ion efflux ATPase	[ 59– 62]
CueR	Cu ^+^, Ag ^+^, Au ^+^	*E*. *coli*	Copper-tolerance genes *copA* and *cueO*	[ 63– 65]
PmtR	Zn ^2+^	*P*. *mirabilis*	A zinc-binding protein	[66]
PbrR	Pb ^2+^	*R*. *metallidurans*	Pb ^2+^-resistance operon *pbrABCD*	[ 48, 67]
ZccR	Zn ^2+^, Cd ^2+^, Co ^2+^	*B*. *pertussis*	A metal ion efflux ATPase	[68]
CadR	Cd ^2+^	*P*. *putida*	*cadA*, a cadmium transporter	[ 50, 51]
CoaR	Co ^2+^	*Synechocystis*PCC 6803	*coaT*, a metal ion efflux ATPase	[69]
NimR	Ni ^2+^	*H*. *influenzae*	Ni ^2+^ uptake transporter (NikKLMQO)	[70]
*Redox-responsive MerR-family TFs*
SoxR	Superoxide	*E*. *coli*	*soxS*, a transcription factor regulating antioxidant genes	[ 71– 74]
NmlR	Formaldehyde	*H*. *influenzae*	*adhC and estD*, detoxification of formaldehyd *e*	[ 75, 76]
*Multidrug-resistance MerR-family TFs*
TipA	Cyclic thiopeptide	*Streptomyces*	Thiostrepton-resistant genes	[ 77– 80]
NolA	Genistein	*B*. *japonicum*	*nodD2*, a transcription factor regulating nodulation genes	[ 81– 83]
BmrR	Multidrug	*B*. *subtilis*	*bmr*, a multidrug-efflux pump	[ 84– 88]
BltR	Multidrug	*B*. *subtilis*	*blt*, a multidrug-efflux pump	[85]
Mta	Multidrug	*B*. *subtilis*	*bmr and blt*, *two* multidrug-efflux pumps	[ 86, 89]
BrlR	c-di-GMP	*P*. *aeruginosa*	*mexAB-oprM* and *mexEF-oprN*	[ 90– 94]
*MerR-family TFs with other functions*
Rv1828	Fatty acids	*M*. *tuberculosis*	Unknown	[95]
Rv3334	Unknown	*M*. *tuberculosis*	*kstR*, a transcription factor regulating lipid catabolism	[96]

These patrol metal sensors also sense overloaded essential metal ions and activate gene programs to maintain their homeostasis. For example, the *E*. *coli* CueR responds to the elevated concentration of cellular free Cu ^+^ and activates the expression of copper efflux ATPase (CopA) and multi-copper oxidase (CueO) [ 63, 97, 98]. *E*. *coli* ZntR maintains the cellular homeostasis of Zn ^2+^ by regulating the expression of a zinc transporter (ZntA) [ 59, 99].

### The redox-responsive MerR-family TFs

Reactive oxygen species (ROS) that induce cellular and genetic damages are produced as an unavoidable consequence of the aerobic lifestyle [100]. The redox-responsive MerR-family TFs are one of the guardians sensing cellular oxidative stress in bacteria [101]. SoxR [71] and NmlR (also named AdhR) [ 75, 102] are two of the currently reported members of the redox-responsive MerR-family TFs. Although SoxR and NmlR fall into the same group, they employ distinct mechanisms to sense oxidative stress. The C-terminal metal binding loop of SoxR coordinates a [2Fe-2S] metal cluster that is proposed to change its overall charge state upon oxidation [ 103– 105]. Once activated, SoxR increases the expression of *soxS* gene, encoding an AraC-family transcription factor that controls the expressions of various antioxidant and damage repair proteins [ 71, 72]. NmlR detoxifies oxidative damages induced by formaldehyde as well as other ROS generators [ 75, 76, 106]. In *H*. *influenzae*, NmlR controls the expressions of AdhC and EstD that work sequentially to convert formaldehyde into formic acid in a glutathione (GSH)-dependent manner [107]. Although biochemical studies suggested that the activity of NmlR is zinc-dependent [75], the crystal structures of apo or DNA-bound NmlR revealed absence of any coordinated metal ions [76].

### The multidrug-resistance MerR-family TFs

The third category of MerR-family TFs is composed of multidrug-resistance receptors that recognize a broad spectrum of exogenous toxic compounds ( Table 1). The representative proteins in this category include TipA [ 77, 78], BmrR [ 84, 85], BltR [ 85, 108], Mta [86], BrlR [ 90– 93, 109], and MrR [110]. TipA has two forms (TipA _L_ and TipA _S_), both of which can sense the self-encoded natural ribosomal inhibitor, thiostrepton [ 77– 79, 111]. Upon forming a covalent bond with thiostrepton, TipA activates the expressions of its own and other proteins necessary for thiostrepton resistance [ 79, 112]. *B*. *subtilis*encodes three members of the MerR subfamily, BltR, BmrR and Mta [ 84– 86]. Mta functions as a master TF that activates the expressions of both multidrug efflux transporters Blt and Bmr; while BltR and BmrR specifically regulate the expressions of the multidrug transporters Blt and Bmr, respectively. BrlR is important for antimicrobial tolerance of *P*. *aeruginosa*by regulating the expressions of the multidrug efflux pumps mexAB-oprM and mexEF-oprN [ 90, 92].

## DNA Recognition of MerR-family TFs

**Figure 3:**
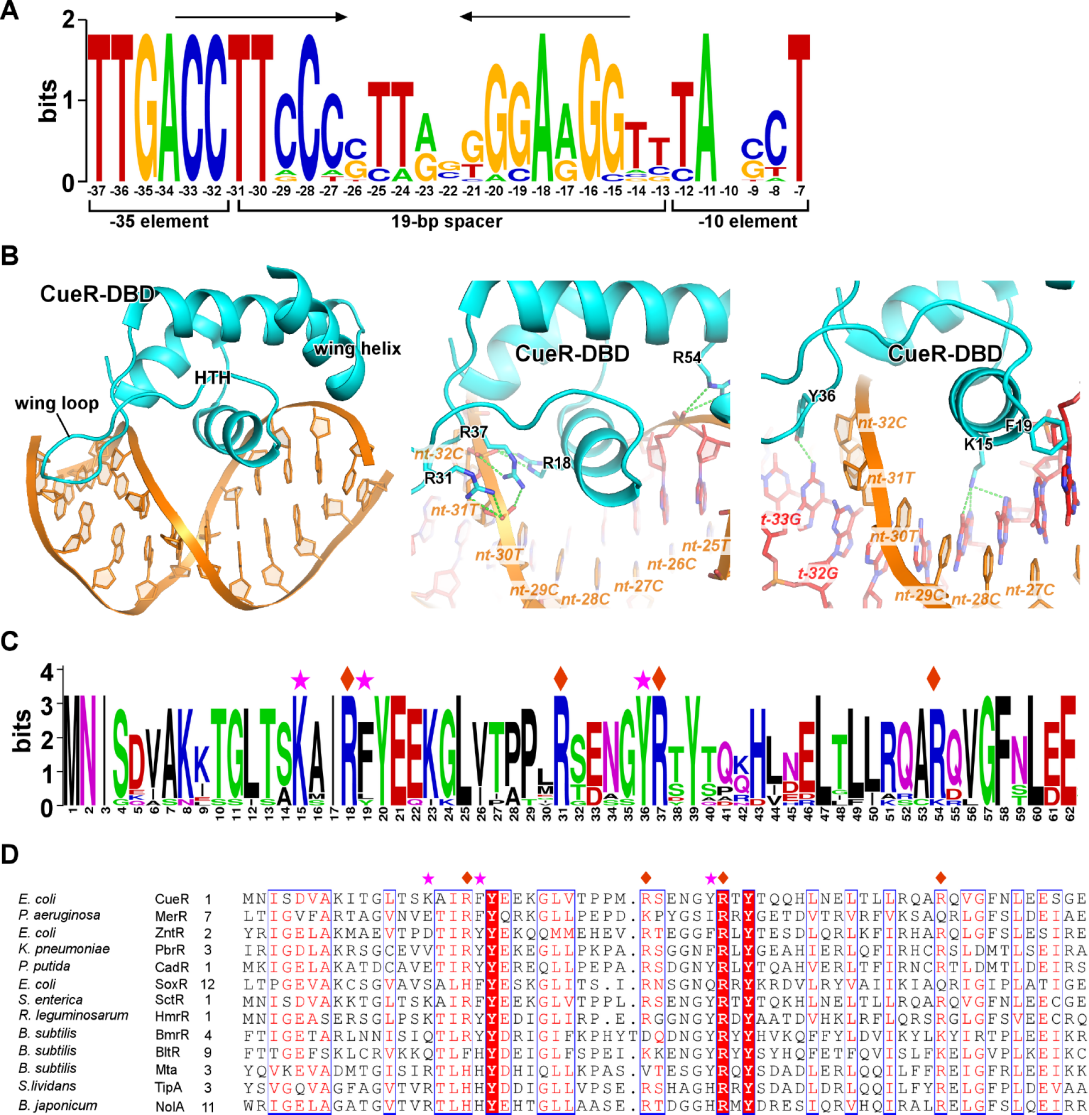
Figure3 **DNA recognition of MerR-family TFs**(A) The consensus DNA sequence logo of E. coli CueR regulated promoters. The palindromic repeats are highlighted by arrows. The positions are numbered respective to the transcription start site (+1). (B) The interaction between the CueR-DBD and dsDNA. The sequence nonspecific interactions between backbone phosphates of DNA and residues of CueR-DBD are shown in the middle panel. The base-specific interactions made by CueR-DBD are shown in the right panel. (C) The consensus protein sequence logo of CueR from various bacterial species. (D) The multiple-sequence alignment of DBD from multiple MerR TFs. The residues contacting backbone phosphates are labeled by diamonds and the residues making base-specific interactions are labeled by asterisks.

The N-terminal winged helix-turn-helix (wHTH) domain of the MerR-family TFs recognizes their cognate long palindromic *cis* elements that are located at the spacer region between the –35 and –10 elements of their regulated promoters [ 51, 64, 73, 76, 87] ( Figure 3A). Both the central helix-turn-helix and two wings (a wing loop and a wing HTH) make direct interactions with DNA. Residues in the central helix and wing loop insert into the major and minor grooves of dsDNA, respectively, and make sequence-specific H-bond and Van der Waal interactions ( Figure 3B). These residues are conserved in homologs of a certain MerR-family TF that recognize the same DNA sequence motifs ( Figure 3C) but vary in MerR-family members that recognize different DNA sequence motifs ( Figure 3D), suggesting that they are responsible for sequence-specific recognition. Meanwhile, several positively charged residues make extensive interactions with the phosphate backbones of DNA and function as clamps to stabilize the protein-DNA interactions during DNA conformational transition. These clamp residues are conserved in the majority of MerR-family TFs, highlighting the importance of these residues, and suggesting that most MerR-family TFs employ the same set of residues to anchor and distort dsDNA ( Figure 3D).

## Signal Reception of MerR-family TFs

In contrast to the conserved wHTH fold of the N-terminal DBD, the C-terminal LBD of the MerR-family TFs varies radically in both length and sequence, conferring the diversity of ligand recognition to this family. In general, the C-terminal LBD of MerR-family TFs is able to coordinate metal cations, sense cellular oxidative condition, and recognizes a broad spectrum of exogenic toxic organic chemicals ( Table 1).

The metal- and redox-responsive MerR-family TFs contain a short C-terminal metal binding loop for coordinating metal ions or metal clusters. The metal-responsive MerR-family TFs exhibit ultra-sensitivity towards their cognate metal ions with reported dissociation constants from micromolar level to nanomolar levels [ 50, 69, 97, 113]. O’Halloran′s group has reported zeptomolar (10 ^–21^ M) and femtomolar (10 ^–15^ M) sensitivity for ZntR/Zn ^2+^ and CueR/Cu ^+^, respectively, under the metal buffering experimental conditions [ 114– 116]. The metal sensors of the MerR family exhibit stringent selectivity towards metal ions of different charge states but are tolerant to ions with the same charge state and the same valence shell configuration.

**Figure 4:**
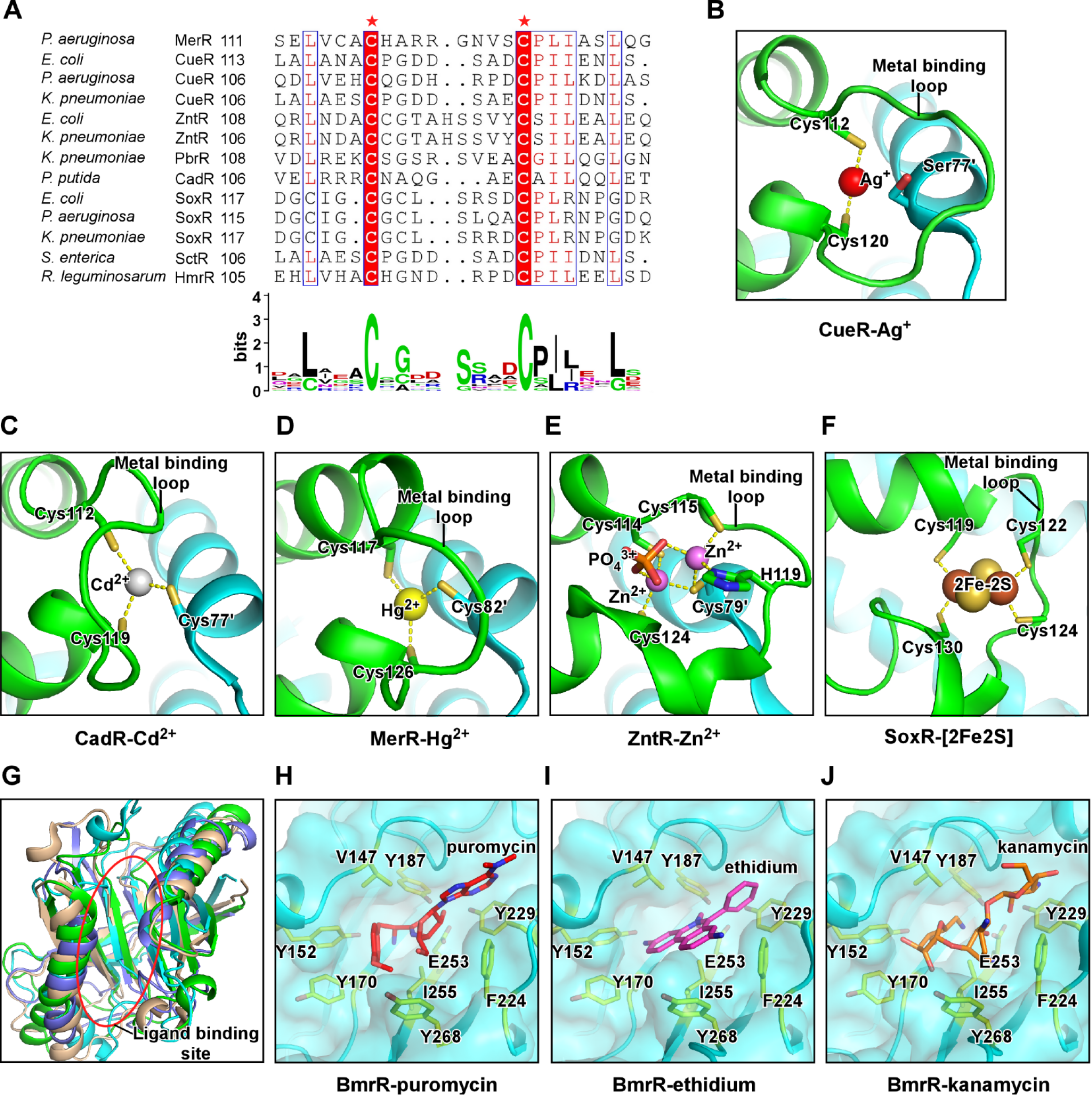
Figure4 **The signal reception of MerR-family TFs**(A) The multiple sequence alignment and consensus protein sequence logo for the ligand-binding domain of MerR-family TFs. The two mostly conserved cysteines are highlighted and labeled by asterisks. (B) E. coli CueR coordinates one molecule of Ag+ through Cys112 and Cys120 of its metal-binding loop. The Ser77’ of the other protomer restrains the conformation of Cys112 (PDB: 4WLW). (C) P. putida CadR coordinates one molecule of Cd+ through Cys112 and Cys119 from one protomer and Cys77’ from the other protomer (PDB: 6JGX). (D) B. megaterium MerR coordinates one molecule of Hg2+ through Cys117 and Cys126 from one protomer and Cys82’ from the other protomer (PDB: 5CRL). (E) E. coli ZntR coordinates two molecules of Zn+ through Cys114, Cys115, H119, and Cys124 from one protomer, Cys79’ from the other protomer, and a phosphate group (PDB: 1Q08). (F) E. coli SoxR coordinates the [2Fe-2S] cluster through residues Cys119, Cys 122, Cys 124, and Cys 130 of the same protomer. (G) Structure superimposition of the ligand-binding domains of four multidrug-resistance MerR TFs, B. subtilis BmrR (cyan; PDB: 3IAO), E. coli EcmrR (green; PDB: 6XLA), P. aeruginosa BrlR (blue; PDB: 5XBT), and E. coli SbmC (light brown; PDB: 1JYH; DNA Gyrase inhibitory protein, Gyrl). (H-J) Detailed presentation of the ligand-binding pocket of B. subtilis BmrR occupied by puromycin (PDB: 3Q3D), ethidium (PDB: 3Q2Y), and kanamycin (PDB: 3Q5R). Residues V147 and I255 server as a hydrophobic pincer. Residues Y152, Y170, Y187, F224, Y229, and Y268 server as an aromatic ring to accommodate drugs with distinct chemical structures. Residue E253 makes auxiliary polar interactions with the drugs.

The C-terminal metal binding loop along with nearby residues creates the metal coordination site that determinates the ion selectivity. Almost all members of the MerR subfamily contain two conserved cysteine residues at both ends of the metal binding loop ( Figure 4A) [114]. The two cysteine residues (Cys112 and Cys120) of *E*. *coli* CueR make two coordinate covalent bonds to Cu ^+^ with an essentially linear S-Cu-S bond angel, while a serine (Ser77) residue from the other protomer inserts into the ion pocket to stabilize Cys112 conformation ( Figure 4B) [114]. The two conserved cysteine residues (Cys112 and Cys119) in *P*. *putida* CadR also participate into the tetrahedral coordination network by making two coordinate covalent bonds with Cd ^2+^, while another cysteine (Cys77′) forms the third tetrahedral coordinate covalent bond ( Figure 4C) [51]. As for MerR, the two conserved cysteines (Cys117 and Cys126 in Tn501 MerR) and the cysteine from the other protomer (Cys82′ in Tn501 MerR) form three coordinate covalent bonds with Hg ^2+^ in a planar trigonal coordination geometry ( Figure 4D) [ 117, 118]. Structural superimposition and sequence alignment suggest that the +1/+2 ion selectivity of metal-responsive MerR-family TFs is mainly determined by the identity of the residue extended from the other protomer (Ser77′ in *E*. *coli* CueR, Cys77′ in *P*. *putida*CadR, or Cys79′ in *E*. *coli* ZntR) that inserts into the ion coordination site [ 51, 114]. It is notable that certain MerR proteins harbor residues that allow coordinating the second ion either at the same site ( *E*. *coli* ZntR) ( Figure 4E) or at a new remote site ( *P*. *putida*CadR) [ 51, 114]. Besides the conserved cysteines residues (Cys119 and Cys130), the C-terminal metal binding loop of *E*. *coli*SoxR contains two more cysteines residues (Cys122 and Cys124) to accommodate a [2Fe-2S] cluster, of which each of the Fe atoms is stabilized by two coordinate valent bonds made by two cysteine residues ( Figure 4F). In summary, MerR proteins respond to metal or oxidative stresses by either directly coordinating cognat metal ions or an oxygen-sensitive metal cluster [2Fe-2S]. The specificity is encoded in the ligand-coordination site, where the number and position of cysteine residues pre-define coordination geometry of their corresponding ligands.

The multidrug-resistance MerR TFs have a Gyrl-like ligand-binding domain (LBD) at their C-terminal domain that is much larger than the metal-binding loop of the metal- and redox-responsive members of the family ( Figure 4G) [119]. Structure superimposition of high-resolution crystal structures of ligand-bound multidrug-resistance MerR proteins reveals that ligands are recognized by a small and rigid pocket of LBD nearby the DNA-binding domain ( Figure 4H–J) [ 87, 93, 110, 120]. The pocket is surrounded by two aliphatic residues functioning as a hydrophobic pincer pair to anchor drugs, a set of aromatic residues allowing docking of drugs with distinct chemical structures, and a trio of acidic residues making auxiliary H-bonds with polar moiety of drugs ( Figure 4H–J) [120]. Besides the common drug-binding pocket in all multidrug-resistance subfamily of MerR proteins, additional pockets were identified in *P*. *aeruginosa* BlrR and *E*. *coli* EcmrR [ 93, 110].

## The Signal-induced Conformational Change

Most MerR-family TFs interact with DNA both in the absence and in the presence of ligands, while the state of ligand occupancy defines the shape of dsDNA and determines the outcome of transcription [ 60, 72, 121, 122]. Recently reported crystal structures of MerR-TF/DNA complex in the activated state all revealed a highly distorted DNA, suggesting a unified mechanism of transcription activation [ 51, 64, 73, 76, 87]. The crystal structures of apo-CueR/DNA (repressive complex) and Ag ^+^-CueR/DNA (activated complex) determined by O’ Halloran’s lab provided excellent opportunity to understand the conformational change induced by ligand binding [64].

The structures show that the C-terminal metal binding loop of apo CueR is disordered but becomes folded upon Ag ^+^ binding. The folded metal binding loop establishes new interaction with nearby structure units and transmits the ligand-biding signal to the distance change of two DBDs. The refolded metal binding loop wedges into the interface of the nearby dimerization helix and DBD. Such event on one hand causes a slight inward rotation of the nearby DBD, and on the other hand causes a small ‘scissors’ movement of dimerization helix resulting in further inward rotation of DBD at the other end. Because both the half palindromic repeats of DNA are tightly anchored by DBD, the conformational change of DBD forces kinking and under twisting of their associated DNA. Other metal-responsive MerR-family TFs likely use the same strategy to trigger the allosteric movement upon metal binding. However, it is still to be determined how the redox-responsive MerR-family TFs, such as SoxR that coordinates the [2Fe-2S] metal cluster and adopts ordered conformation in both the oxidative and reduced conditions, interchanging their conformations [ 73, 101].

The signal-induced conformational change is less determined for multidrug-resistance MerR TFs, as structural information for direct comparison of TF/DNA complexes in the absence and presence of ligand is unavailable. The crystal structures of drug-bound BmrR-DNA complexes and cryo-EM structures of *E*. *coli* EcmrR-RPo revealed direct interaction between DBD and LBD, suggesting that drug binding might affect the LBD-DBD interaction leading to DNA distortion through an unknown signal transmission manner [ 87, 110].

## Transcription Repression by MerR-family TFs

The gene promoters regulated by MerR-family TFs typically possess abnormally long spacers (19–20 bp) between the –35 and –10 elements compared with the optimal length (17±1 bp) ( Figure 3A) [7]. The unique long spacer is essential for regulation, as shortening the spacer renders promoters irresponsible to activation by their cognate MerR TFs [ 123– 125]. The strict requirement of the –35/–10 spacer length (17±1 bp) is defined by the distance of σ _4_ and σ _2_, which are anchored near the RNA exit channel and the top of RNAP main cleft, respectively [ 18, 19]. The unique structure architecture of RNAP-σ ^70^ holoenzyme allows sequential recognition of the –35 and –10 elements of bacterial gene promoters containing optimal spacer length [30]. Increase of spacer length in 2–3 bp results in increase of the –35/–10 element distance in 6.8–10.2 Å and rotation in 72°–108° around the helix axis [64]. Consequently, the –10 element of promoter DNA, when its –35 element is bound by σ _4_, is extended and rotated away from the σ _2_ domain, preventing further DNA unwinding and resulting in a very low basal transcription activity.

Binding of apo MerR-family TFs further inhibits the weak basal transcription activity of their regulated promoter DNA [ 64, 65]. The crystal structure of apo CueR-DNA shows that the engagement of apo CueR shifts the trajectory of promoter dsDNA further away from the σ _2_ domain and restrains the dsDNA in the inactive shape [64]. Although the apo CueR-dsDNA is the only reported crystal structure of apo MerR-TF/DNA, footprinting data support that other metal-responsive MerR TFs probably also interact with a straight dsDNA in the absence of ligand binding [ 121, 126].

## Transcription Activation by MerR-family TFs

**Figure 5:**
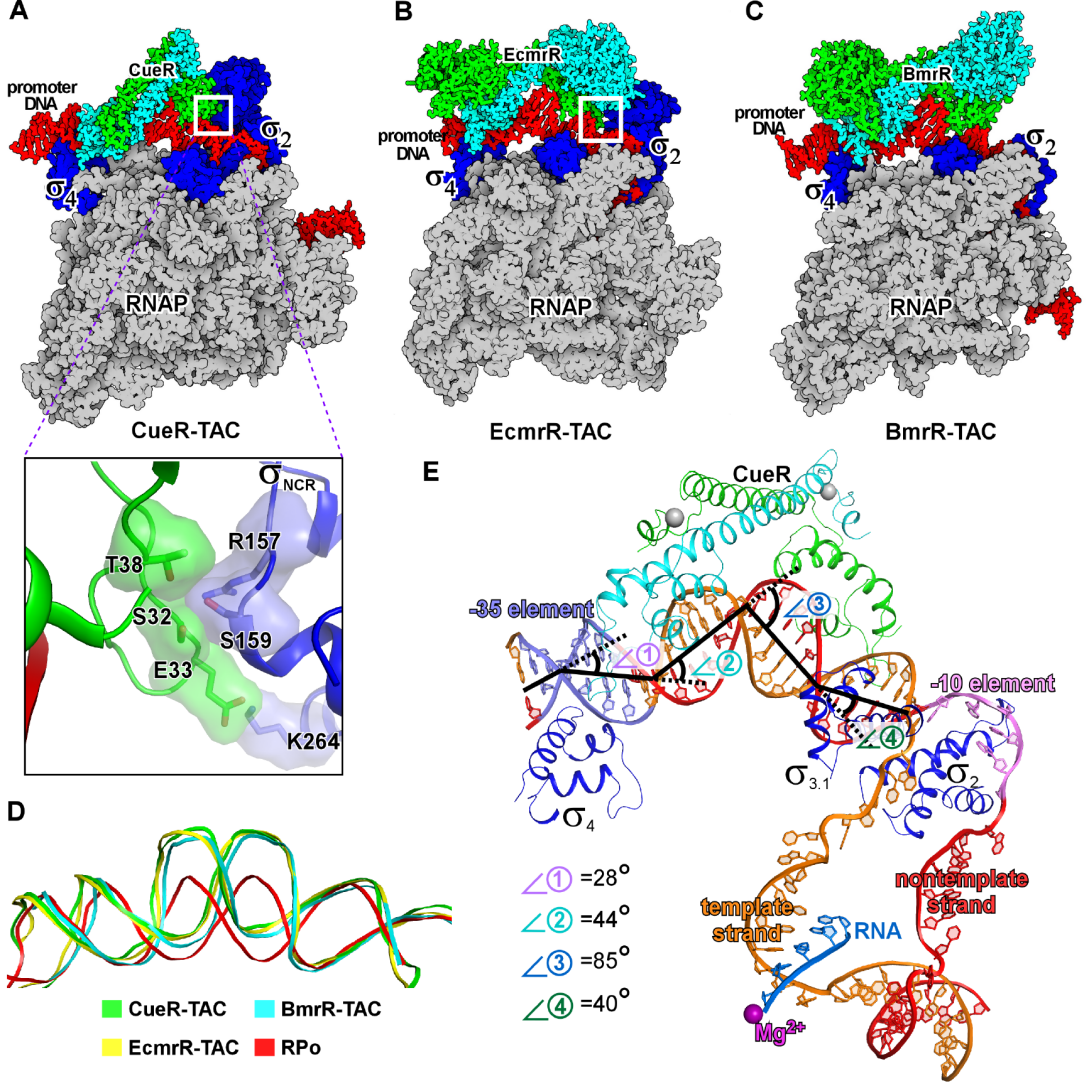
Figure5 **The cryo-EM structures of transcription activation complexes comprising MerR-family TFs**The cryo-EM structures of (A) E. coli CueR transcription activation complex (PDB: 6XH7), (B) E. coli EcmrR transcription activation complex (PDB: 6XL5), and (C) B. subtilis BmrR transcription activation complex (PDB: 7CKQ). The insertion box shows the small surface patch of CueR-DBD that interacts with the σNCR. (D) Structure superimposition of the upstream dsDNAs of the above three transcription activation complexes and that of a bacterial RPo (PDB: 6OUL). (E) The kinks of the upstream promoter DNA at positions −35 (⦟1, 28°), −30 (⦟2, 44°), −24 (⦟3, 85°) and −18 (⦟4, 40°) in the cryo-EM structure of E. coli CueR-TAC (PDB: 6LDI). Kink 1 at −35 is induced by CueR and σ704, and kinks 2, 3 and 4 are induced by the CueR dimer.

The long-term debate regarding the transcription activation mechanism of MerR-family TFs is whether and what extent the MerR TF-RNAP interaction contributes to transcription activation [ 7, 121, 127]. The canonical Class I and Class II transcription activators bridge RNAP and promoter DNA by making bipartite interaction; the interaction with RNAP is necessary as mutating the activator-RNAP interface substantially reduces transcription activation activity [37]. However, the concept of requirement of TF-RNAP interaction for transcription activation is challenged by recent cryo-EM structures of transcription activation complexes comprising MerR-family TFs, *E*. *coli* CueR-TAC, *B*. *subtilis* BmrR-TAC, and *E*. *coli* MrR-TAC ( Figure 5A–C) [ 65, 88, 110, 128].

In these structures, the MerR-family transcription factors reside on one face of the upstream promoter DNA, while the RNAP-σ ^70^ (or RNAP-σ ) holoenzyme recognizes the -35 and -10 elements from the opposite face. The architecture explains the paradox that MerR-TFs activate transcription while occupying the core promoter region, an interaction typically represses transcription due to steric hinderance between TFs and RNAP. Intriguingly, although RNAP and MerR-TF occupy the same core promoter regions, BmrR makes interaction with neither RNAP core enzyme nor σ _A_ factor in the cryo-EM structure of *B*. *subtilis* BmrR-TAC [88], while *E*. *coli* CueR and EcmrR only contact a small surface patch of the non-conserved region of σ _70_ factor (σ _NCR_) in the cryo-EM structures of *E*. *coli* CueR-TAC and *E*. *coli* EcmrR-TAC ( Figure 5A) [110]. The TF-σ _NCR_ interaction is not essential for their transcription activation activity, because MerR TFs (CueR and ZntR) in *E*. *coli* are able to activate transcription of their regulated promoters using evolutionarily distant RNAPs ( *i*. *e*. RNAPs from *M*. *tuberculosis*, *T*. *thermophilus*, and *S*. *coelicolor*) that have completely different σ _NCR_ domains [65]. The TF-σ _NCR_ interactions observed in *E*. *coli* CueR-TAC and EcmrR-TAC, however, contribute to the transcription activation by providing auxiliary bridge interaction between RNAP and DNA [ 110, 128].

**Figure 6:**
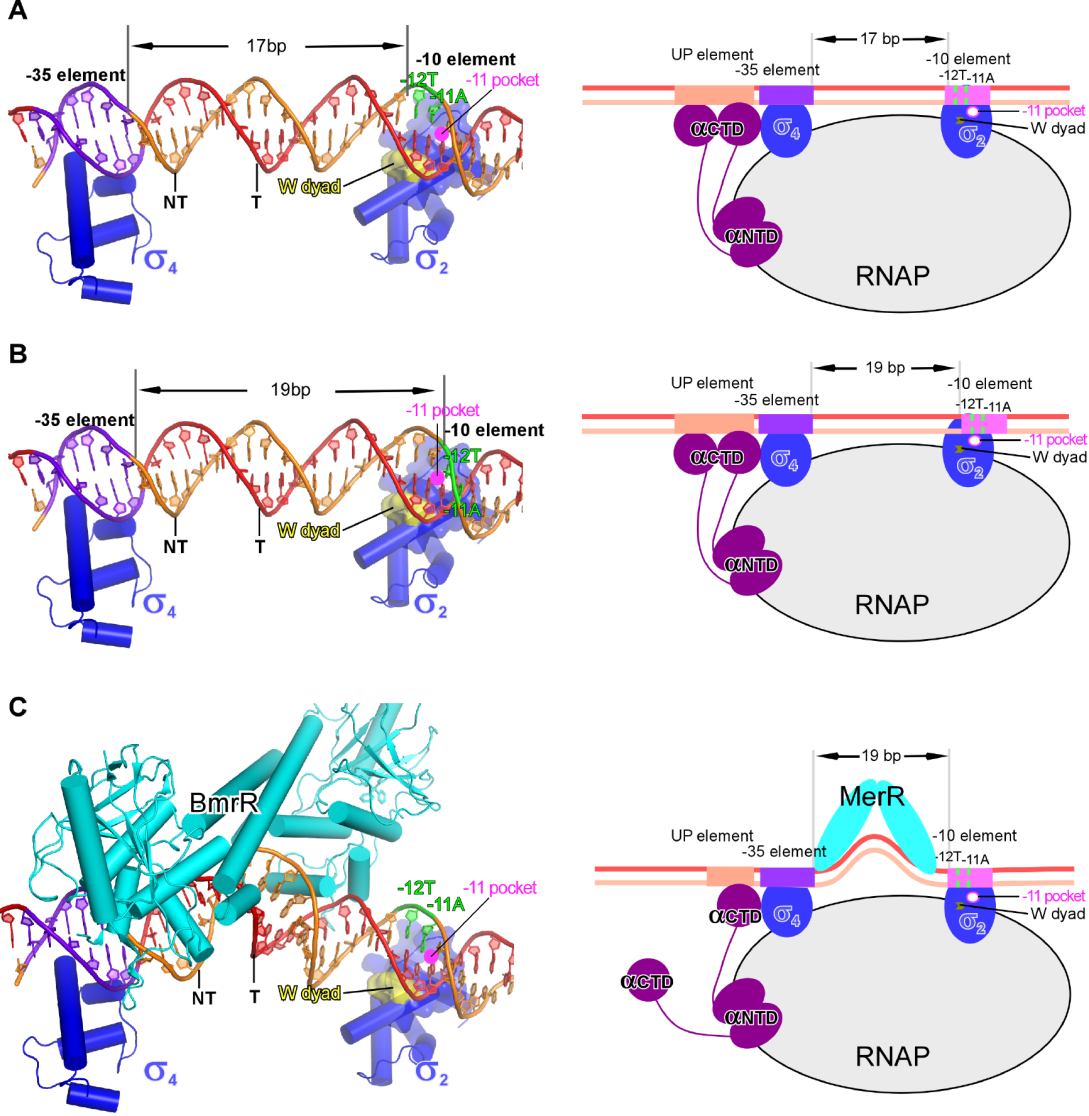
Figure6 **The DNA-distortion mechanism of transcription activation by MerR-family TFs**(A) The –35 and –10 elements are properly aligned on the protein surface of σ4 and σ2. The –11A of the nontemplate strand DNA is close to the –11 pocket. The panel figure is prepared from a B. subtilis RPc model comprising a promoter DNA with 17-bp –35/–10 spacer. (B) The –11A of the nontemplate strand DNA is rotated away from the –11 pocket in the B. subtilis RPc model comprising a promoter DNA with 19-bp –35/–10 spacer. (C) The BmrR-induced central kink realigns the −35 and −10 elements to a proper space and phase for simultaneous engagement by σ4 and σ2 according to the structure model of a BmrR-RPc complex. Yellow surfaces (left) and arrows (right) show the tryptophan dyad; pink circles show the –11 pocket.

Several lines of evidence point to a ‘DNA distortion’ mechanism of transcription activation by the MerR-family TFs. The DNA distortion induced by ligand-bound MerR was initially proposed based on results of DNA mobility and footprinting assays [126], and subsequently directly visualized in crystal structures of ligand-bound MerR-family TFs complexed with DNA, including crystal structures of CueR-Ag ^+^/DNA, CadR-Cd ^2+^/DNA, SoxR-[2Fe-2S]/DNA, and NmlR-Ni ^2+^/DNA [ 51, 64, 73, 76, 87]. The most striking feature of the distorted DNA is the 90° kink at the center of the palindromic dyad, where the two central base pair steps are often broken and the minor groove becomes significantly wider even than the canonical major groove, resulting in a A-DNA-like structure ( Figure 5D). The distal ends of their cognate dsDNA are still gripped by the wHTH domains, resulting in a ‘Ω’-like shape of the dsDNA ( Figure 5D,E). Such conformational switch of dsDNA results in pronounced changes both in the distance and in the phase angle between the -35 and -10 elements. Detailed comparison between the canonical B-formed dsDNA and activator-bound dsDNA revealed that the DNA distortion shortens the –35/–10 distance by ~7Å and reduces the phase angles of dsDNA by 72°, close to those of a 17-bp spacer promoter ( Figure 6) [64].

In summary, the structural and the biochemical evidence provides strong support for the DNA distortion paradigm of allosteric transcriptional control by the MerR-family TFs. Such mode of transcription activation doesn’t necessitate the RNAP-TF interaction, and thus is distinct from the transcription activation mechanism of canonical Class I and Class II transcription activation modes.

## Conclusion

This review discussed the studies of the past years, regarding the unique mechanism of transcription activation of the MerR-family TFs with focus on the structural and biochemical data. Intriguingly, a conceptional similar DNA-distortion mechanism has also been employed by the eukaryotic TATA-box binding protein (TBP) during eukaryotic polymerase II transcription initiation. A recent study in Seok’s lab identified FruR, a GalR-LacI family transcription factor in *V*. *cholerae*, which binds to its *cis* element located between the -35 and -10 element with a 20-bp spacer and likely uses a similar DNA-distortion mechanism to regulate the expressions of its target genes [129]. These discoveries suggest that such DNA-distortion mechanism might be more widely employed by eukaryotic and prokaryotic transcription factors than we expected. The unique RNAP-contact-independent action, the ultra-sensitivity and selectivity towards cognate ligands, and the stringent transcription regulation make the MerR-family TFs ideal transcription modules in synthetic biology uses.
